# Post Traumatic Stress Disorder : Clinical description of 101 children

**DOI:** 10.1192/j.eurpsy.2022.1062

**Published:** 2022-09-01

**Authors:** S. Bourgou, K. Nourchene, H. Rezgui, A. Belhaj

**Affiliations:** 1 hospital of mongi slim, Pedopsychiatry, Tunis, Tunisia; 2 Razi hospital, Pedopsychiatry, Tunis, Tunisia

**Keywords:** clinical manifestation, description, post traumatic stress disorder, Children

## Abstract

**Introduction:**

Post traumatic stress disorder (PTSD) is a mental health condition that’s triggered by a psychotraumatic event .

**Objectives:**

The aim of our study was to investigate the clinical manifestations of PTSD in the pediatric population.

**Methods:**

This is a descriptive cross-sectional study carried out on children over the age of 7 and victims of a traumatic event that had occurred at least one month before. They were recruited from August 2020 to April 2021, in child psychiatry department. The clinical manifestations were evaluated using the Clinician Administered PTSD Scale Child and Adolescent version for DSM 5 (CAPS CA5) in Tunisian dialect. The statistical processing of these data was carried out using SPSS 26 software.

**Results:**

We recruited 101 children who had experienced a traumatic event which was in 35.6% physical assault, 47.5% sexual assault and in 16.8 % exposure to death. The mean age was 10.7 years at the onset of traumatic event and 11.74 years at the interview. We noted in our patients a female predominance at 64.4%. Diagnosis of PTSD according to the diagnostic criteria of the DSM5 was retained in 54.5% of cases. Intrusive symptoms were present in 81.2%, with 66.3% of involuntary, intrusive memories. Persistent avoidance of stimuli was noted in 80.2%. 71.3% of cases suffered from negative cognition and mood. We found in 66.3% marked alterations in trauma-related arousal and reactivity. Resulting in clinicialy significant distress in71.3%.

**Conclusions:**

Recognizing PTSD symptoms is essential for diagnosis to initiate specialized care and reduce impairment at this critical age.

**Disclosure:**

No significant relationships.

